# Collision Avoidance and Stability Study of a Self-Reconfigurable Drainage Robot

**DOI:** 10.3390/s21113744

**Published:** 2021-05-28

**Authors:** Rizuwana Parween, M. A. Viraj J. Muthugala, Manuel V. Heredia, Karthikeyan Elangovan, Mohan Rajesh Elara

**Affiliations:** 1 Engineering Product Development, Singapore University of Technology and Design, 8 Somapah Road, Singapore 487372, Singapore; viraj_jagathpriya@sutd.edu.sg (M.A.V.J.M.); e_karthikeyan@sutd.edu.sg (K.E.); rajeshelara@sutd.edu.sg (M.R.E.); 2 Apt of Engineering and Technology, Universidad Autónoma de Occidente, Macario Gaxiola and Highway Mexico 15, Los Mochis 81223, Sinaloa, Mexico; manuel.vega@udo.mx

**Keywords:** drain robot, reconfigurable robot, level-shifting, stability, collision avoidance

## Abstract

The inspection and maintenance of drains with varying heights necessitates a drain mapping robot with trained labour to maintain community hygiene and prevent the spread of diseases. For adapting to level changes and navigating in the narrow confined environments of drains, we developed a self-configurable hybrid robot, named Tarantula-II. The platform is a quadruped robot with hybrid locomotion and the ability to reconfigure to achieve variable height and width. It has four legs, and each leg is made of linear actuators and modular rolling wheel mechanisms with bi-directional movement. The platform has a fuzzy logic system for collision avoidance of the side wall in the drain environment. During level shifting, the platform achieves stability by using the pitch angle as the feedback from the inertial measuring unit (IMU) mounted on the platform. This feedback helps to adjust the accurate height of the platform. In this paper, we describe the detailed mechanical design and system architecture, kinematic models, control architecture, and stability of the platform. We deployed the platform both in a lab setting and in a real-time drain environment to demonstrate the wall collision avoidance, stability, and level shifting capabilities of the platform.

## 1. Introduction

In recent decades, the development of hybrid locomotion robots for various applications has increased. The combination of two or more kinds of locomotion increases the flexibility to reach a displacement goal given the needs of society, i.e., the NASA Athlete robot [1] for space exploration and robots used for defence, including [2], the lunar Rover Wheel-Leg Foot robot [3], or the hybrid robot KaMERO with expressive body motions [4]. Some robots use hybrid locomotion to develop novel kinds of displacement for robotics, like the jumping robot Airhopper [5].

In the same way, the interest in the study of areas of difficult access for human beings has been increasing, thus, giving rise to various robot platforms, such as: pipe inspection robots [6] with multiple kinds of hybrid locomotion [7] for areas where humans cannot access [8]. The rolling, crawling, and climbing robot Scorpio used for even and uneven terrains [9] can climb vertical surfaces [10]. Some hybrid robots have complex locomotion inspired from insects [11] due to the capacity of insects to reach complicated areas [12]. One of the topics of the greatest interest and focus of this document is the analysis and maintenance of drainage areas.

**Figure 1:**
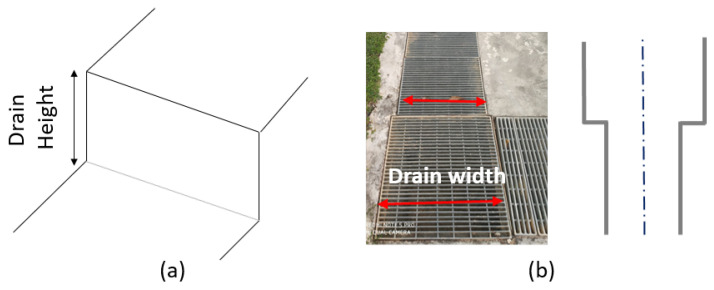
(**a**) Morphology of a drain with a level shift. (**b**) Drain width variation.

Some robot platforms developed for this specific application include a municipal drainage dredging robot [13], a drainage pipelines dredging robot [14], drain pipe inspection robots [15], and under water robots [16]. Drains exist in different designs as shown in Figure 1. The ranges of the height and width of these drains are 0.3 to 1.1 m and 0.2 to 0.8 m, respectively [17]. The drain water flow is very important due to flood risk or vegetation growth [18], and, at the same time, there are mosquito [19] and water analysis studies [20].

Navigation in uneven terrain or terrains with different levels demands control behaviours, e.g., a load leg/wheel load control [21] and kinematics control for a wheeled robot [22]. The control for these robots requires stability analysis during level shifting [23,24], controlling the motion according to the environment [25] and the step-passing [26], adapting to the terrain considering the robot’s capabilities [27]. The objective of this paper is to show the development of a robot for drains with an uneven height with many level shifts and bends that are inaccessible to humans. During operation, the robotic platform should have static and dynamic stability.

Apart from the consideration of stability, a robot intended for the inspection of a drain should have the ability to navigate safely without collisions. The space availability for a robot in a drain is confined since most of the drains are narrow in shape. The unpredictable variation of the surface roughness of a drain might hinder the smoothness of the navigation of a robot [28,29]. Furthermore, sensory information noise further degrades the navigation in this sort of unknown environment [30,31]. Therefore, the realization of safe and smooth navigation in a drain is difficult. A robot designed for inspecting drains should also have the ability to facilitate safe navigation in a drain by avoiding possible collisions with the sidewalls.

Much literature has exploited the uncertainty handling capabilities of fuzzy logic in diverse application domains of robots, including climbing robotics [32,33], autonomous vehicles [34,35], aerial robotics [36,37], and maintenance and inspection robotics [38,39]. In particular, fuzzy logic has often been utilized for navigating robots in unknown environments. In this direction, [40] proposed an interval type-II fuzzy logic system for establishing a global path planning method for an indoor mobile robot. The proposed method is capable of performing point-to-point navigation of a robot while avoiding obstacles through overhead camera-based visual-servoing.

The performances of point-to-point navigation by learning-based fuzzy logic systems and a fuzzy logic system pruned by human expertise were compared in [41]. According to the outcomes of the comparison, the fuzzy logic system pruned based on human experience outperformed the proposed learning-based fuzzy logic system. Similarly, much work conducted for the point-to-point navigation of mobile robots while avoiding obstacles can be seen in the literature [42,43,44,45,46].

Apart from point-to-point navigation methods, wall-following methods for navigating robots within indoor environments have also been developed [47,48,49,50]. The majority of the work on the development of fuzzy logic systems for mobile robot navigation is limited to tasks, such as point-to-point navigation and wall-following inside indoor environments. However, the above-cited fuzzy logic systems cannot be adopted for the safe navigation of a reconfigurable robot intended for drain inspection.

With these design considerations, a quadruped drain inspection robot, named Tarantula-II, was developed [51]. In the previous study, the detailed mechanism of the platform is described. The objective of this paper to analyse the stability during the level shifting process and the collision avoidance performance of the robot’s body within the side walls of the drain. Cyber security and internet of things based models are being widely adapted in industrial vehicles to prevent data leakages, sensor hacking, and remote manipulation [52,53,54,55]. These models will be implemented to the autonomous version of the robotic platform in future work to avoid the risk of intrusion attacks on the robot.

The paper describes robot architecture, including the mechanical design, electronics layout, and software architecture of the platform. Section 3 and Section 4 describe the kinematics formulation, and stability analysis of the robot platform, respectively. Section 5 explains the algorithm of the control for wall collision avoidance. Section 6 presents real-time monitoring of the platform stability and wall collision avoidance. Finally, the paper is concluded in Section 7.

## 2. Platform Architecture

**Figure 2:**
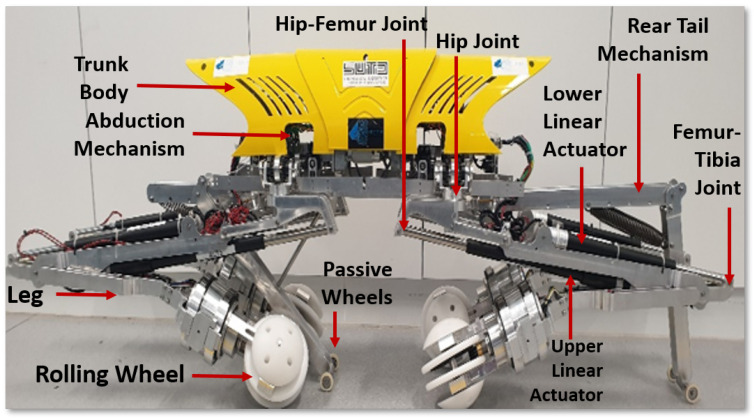
Tarantula-II Platform showing the different parts.

This section explains the Tarantula-II robot’s mechanical design, electronics layout, and navigation process. The Tarantula platform has a weight of 20 kg and is 1.2-m long in total. The longitudinal spacing between the front and rear legs has a fixed length of 0.8 m. The platform has a minimum and maximum height of 0.5 to 1 m. The platform primarily consists of three modules, namely the trunk body, legs, and wheels, as shown in Figure 2. The trunk body is design to be placed parallel to the drain section, and the trunk cover is made of four pieces that are fabricated using high-grade plastics to reduce the weight.

**Figure 3:**
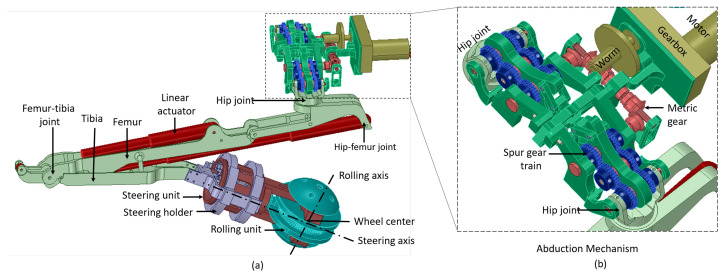
A CAD model of the Tarantula-II Platform showing (**a**) the leg and wheel and (**b**) the simultaneous abduction mechanism.

The trunk accommodates a suspension mechanism that supports the electronics module and an abduction mechanism. Each leg has three parts, the hip, femur, and tibia, as shown in Figure 3a. The hip is connected to the platform’s trunk with the hip joint, which provides abduction and adduction movement to each leg. Each femur module consists of two linear actuators, with two revolute joints at the proximal and distal end. The distal end of the tibia is connected to the rolling wheel, and this is called the ankle joint.

Figure 3b shows the simultaneous abduction module of the Tarantula-II platform, which is placed on a suspension system that supports components by absorbing the shock and vibration. The trunk consists of two abduction units one at the anterior and another at the posterior side of the platform. Each abduction unit consists of a set of gear trains that is driven by a DC motor. Each abduction unit provides 0 to 180 degrees of rotation of both front legs about the abduction axis in the frontal plane. This results in varying the wheel spacing between the front legs. Similarly, the posterior abduction mechanism rotates both the rear legs and modulates the wheel spacing. Both motors synchronously move both the front and rear legs and maintain equal wheel spacing between the wheels.

Figure 3 shows the wheel module that consists of a rolling and steering wheel units, which are driven independently. Each wheel has the capability to roll along two different axes (rolling and steering). The rolling wheel unit consists of four distinct modular parts made up of Delrin material. This unit is driven by a DC motor that provides forward and backward movement to the platform. The entire steering unit is made up of aluminum with the proximal end placed inside spacing of the rolling unit. The distal end of the steering unit is attached to another DC motor that provides rotation about the steering axis and, hence, results in lateral movement of the platform. The details of the self-reconfigurability, structural analysis, and kinematics of each leg and wheel are described in our published work [51].

**Figure 4:**
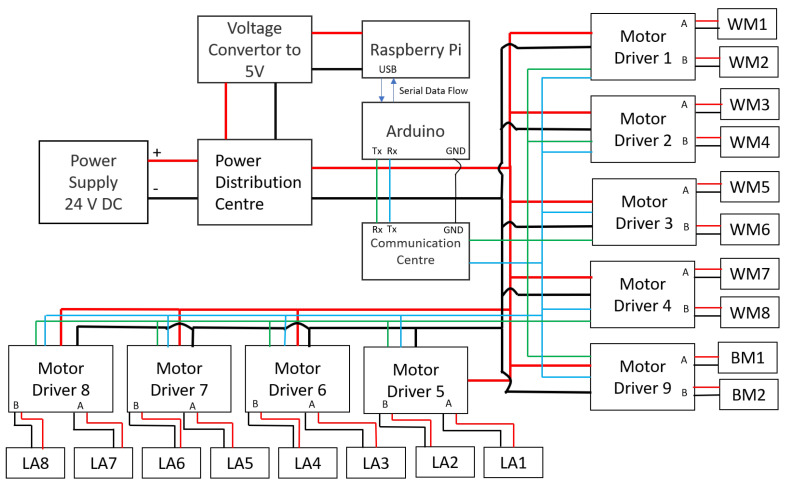
System architecture of the platform.

Figure 4 shows the electronics layout of the platform. Tarantula employs eight linear actuators (LAs), four wheels, two body motors, and the abduction principle to achieve locomotion. The body motors are used for the abduction movement. Each wheel unit has a pair of DC motors that provides motion along two different directions. These two motors are controlled by a single Roboclaw driver. Each leg has a pair of linear actuators, which are responsible for changing the leg height. In total, there are eight motors responsible for the wheel locomotion. All the legs have eight linear actuators and each actuator controls the angle of rotation of the joints.

**Figure 5:**
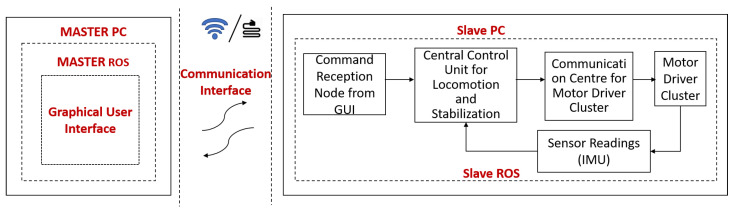
Communication architecture of the platform.

The platform’s trunk consists of a single-board computer and a micro-controller to actuate all the abduction motors, linear actuators, and wheel motors. Each motor driver consists of a unique hexadecimal address through which the control commands can be identified and sent. The transmission and reception pins of all the motor drivers are connected in parallel under a communication centre as shown in Figure 5. The entire system runs on the ROS framework.

The operator sends the command through a mainframe, which is termed as a master module in our case, and the respective command is received by the SBC, slave module, on the robot side. This slave module then issues a corresponding control command to a microcontroller board that is sharing the same transmission and reception lines with the motor drivers. The motor driver drives the motors, and the necessary feedback will be sent back to the slave module by the microcontroller for further decision making.

The master and the slave module communicate over a LAN (local area network) cable, while the slave module and the microcontroller communicate over a serial bus. The SBC is powered up by a 5 V voltage regulator, while the motor drivers are powered from the lines that are drawn from an onboard 24 V DC power distribution centre. The robot platform consists of a single camera, which is mounted on the anterior side of the trunk. The robot records images and the legs are not in the way of the captured images. The robot also uses IR sensors for collision avoidance among the drain wall and an IMU sensor for achieving stable postures.

## 3. Platform’s Kinematics

The proposed kinematics modelling is used to determine the robot position and orientation based on the wheel rotation measurements. The Tarantula platform is a four wheeled robot with an independent drive for each wheel. For navigation in the confined drain and avoiding wall collisions, we implemented a simplified kinematic equation that was based on differential wheel drives. Each set of wheels on the left (front left $W_{FL}$ and rear left $W_{RL}$ wheel) and right (front right $W_{FR}$ and rear right $W_{RR}$ wheel) sides are represented by a virtual wheel, $W_{L}$ and $W_{R}$, respectively.

**Figure 6:**
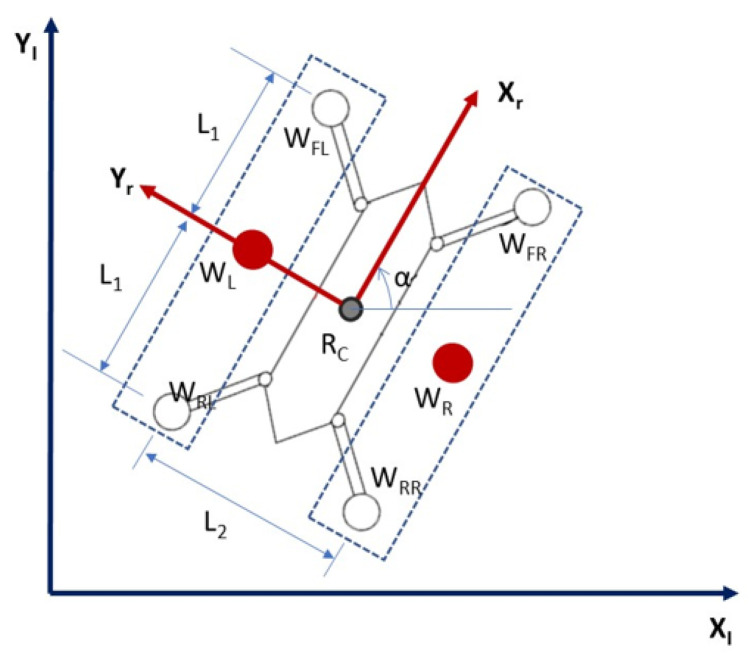
Schematic diagram of the wheel layout of the platform.

The robot can change its direction by varying the relative rate of rotation of these virtual wheels. Figure 6 shows the schematic diagram of the wheel layout, which is used for kinematics derivation. $l_{1}$ is the distance between the robot centre and front (rear) wheels. $l_{2}$ is the distance between the robot’s left and right virtual wheels. *r* is the wheel radius. $\phi_{L},\omega_{L},$ and $v_{L}$ are the angular position, angular velocity, and linear velocity of the virtual left wheel. $\phi_{R},\omega_{R},$ and $v_{R}$ are the angular position, angular velocity, and linear velocity of the virtual right wheel. $\alpha ,\dot{\alpha }$ are the angular position and angular velocity of the robot platform. $x_{c}$, $y_{c}$ are the components positions of the platform. The following equations govern the kinematics relations.

(1) $$\begin{array}{cc} \omega_{L}= & \frac{\omega_{FL}+\omega_{RL}}{2}\\ \omega_{R}= & \frac{\omega_{FR}+\omega_{RR}}{2} \end{array}$$

(2) $$\begin{array}{cc} v_{L}= & r\omega_{L}\\ v_{R}= & r\omega_{R} \end{array}$$

(3) $$\begin{array}{cc} \alpha = & \left(\phi_{R}-\phi_{L}\right)\frac{r}{l_{2}}\\ x_{c}= & \int_{o}^{t}\left(v_{L}+\frac{\dot{\alpha }l_{2}}{2}\right)\cos\alpha \;dt\\ y_{c}= & \int_{o}^{t}\left(v_{L}+\frac{\dot{\alpha }l_{2}}{2}\right)\sin\alpha \;dt \end{array}$$

## 4. Stability Analysis

**Figure 7:**
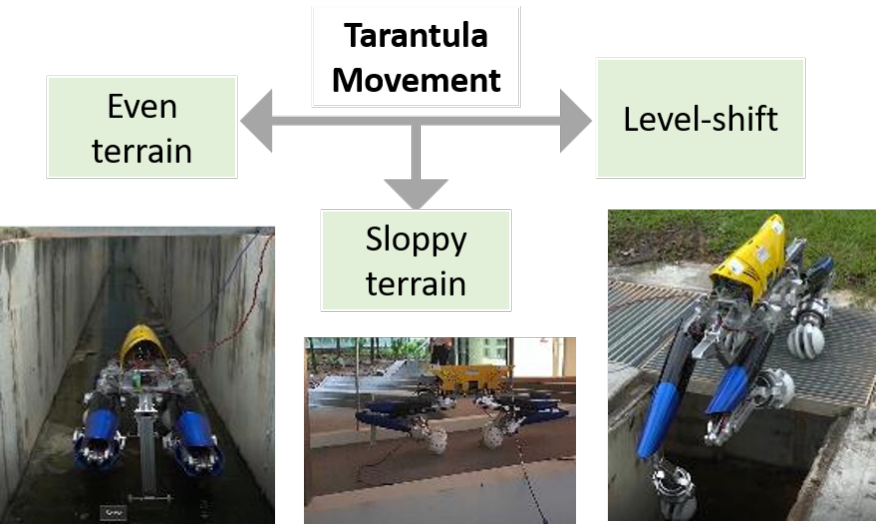
Three distinct types of locomotion for the Tarantula-II platform.

The locomotion of the Tarantula II platform is classified as movement on flat terrain, sloppy terrain, and level shifting as shown in Figure 7. The platform navigates at lower speed on flat and sloppy terrain. Despite the low speed conditions, the static and dynamic balance of the platform are to be maintained during navigation and level shifting. The platform’s stability depends on the leg height, the terrain conditions, speed, contact point on the ground, and navigation control. The research community has been using the qualitative parameters, including the static stability margin, longitudinal margin, crab longitudinal margin, energy stability criterion, and tip over energy stability criterion [56,57,58,59,60], to evaluate the stability of the quadruped moving at low speed.

McGhee et al. introduced the concept of a static stability criterion for a machine walking at constant speed over flat and even terrain and uneven terrain [61,62]. The support polygon is defined as the horizontal projection of the contact point. The Static Stability Margin is defined for a given support polygon as the smallest of the distances from the centre of mass projection to the edges of the support polygon. As per this criteria, the platform is statically stable if the horizontal projection of its centre of mass is inside the support polygon.

**Figure 8:**
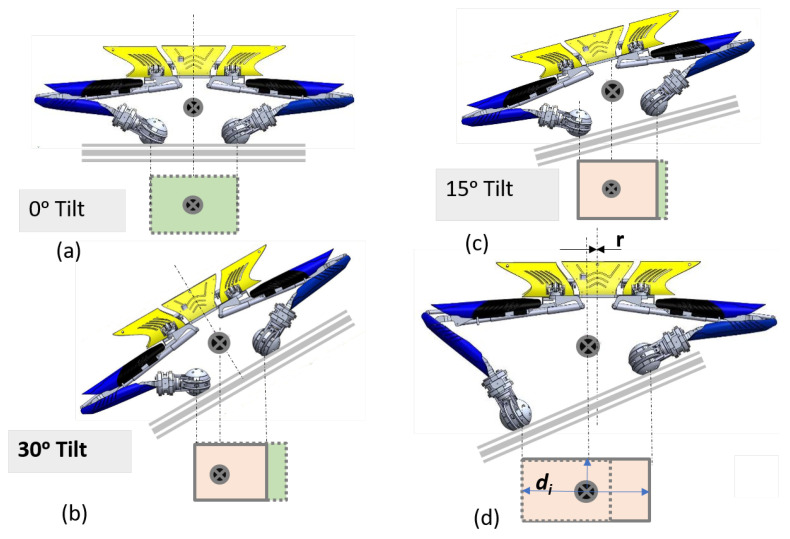
The variation of the CG (centre of gravity) location of the platform on (**a**) flat surface, (**b**) trunk parallel to an inclined plane of 15 degree tilt, (**c**) trunk parallel to an inclined plane of 30 degree tilt, and (**d**) trunk in horizontal position on an inclined plane of 30 degree tilt.

For navigation of the robot inside the drain without falling, the robot’s centre of gravity (CG) needs to be actively shifted to maintain an equilibrium position. Thus, it is required to manage the balance of the platform during locomotion on sloppy terrain and level shifting tasks. During locomotion, the controller helps all the wheels to make contact with the terrain. During level shifting, a minimum of three legs are needed to support the whole body. The concept of a support polygon has been applied to study the stability of the platform. The rectangle below the Tarantula platform in Figure 8 represents the support polygon, which is the projection of the contact locations of the wheel on the ground.

On a horizontal plane (zero degree tilt), when all the legs are of the same length and the trunk is perfectly horizontal, the centre of gravity is exactly at the centre of the support polygon. As we move the platform on the inclined plane from 0 to 30 degrees with the trunk body parallel to the plane, the area of the support polygon gradually reduces, and the centre of gravity shifts towards left. With further increases in the tilting angle and keeping the trunk body parallel to plane, the area of the support polygon becomes further reduced, and there is a high chance that the centre of gravity may move out of the support polygon.

Thus, in the Tarantula platform, we intended to adjust the leg height and to keep the trunk body horizontal irrespective of the inclination. As a result, the area of the support polygon increases, and the centre of gravity is always in the centre. If the $d_{i}$ is the distance of the CG location from the nearest support side of the support polygon, the stability margin is $r_{i}$, which is the shift in the CG location. The horizontal position of the trunk on the inclined to maintain the magnitude of $r_{i}$ less than $d_{i}$.

**Figure 9:**
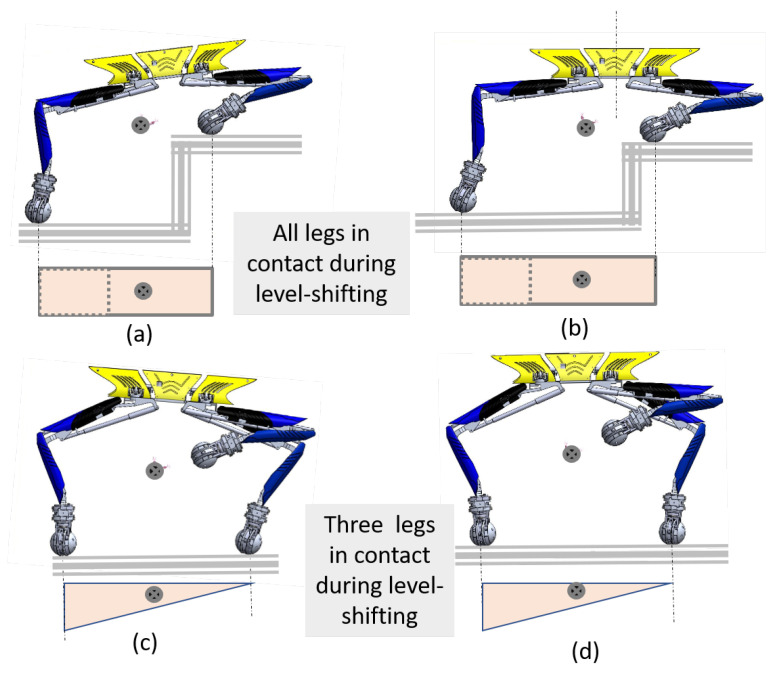
The variation of the CG location and the support polygon of the platform during level shifting: (**a**) all legs are in contact and the trunk is inclined, (**b**) all legs are in contact and the trunk is horizontal, (**c**) three legs are in contact and the trunk is inclined, and (**d**) three legs are in contact and the trunk is horizontal.

During level shifting, when all four legs are in contact with the ground, the support polygon is a rectangular area, as shown in Figure 9a,b. Depending upon the tilt of the trunk, the CG location shifts. However, in both cases, the CG lies inside the support polygon. During level shifting, when three legs are in contact, the support polygon is a triangular area as shown Figure 9c,d.

**Figure 10:**
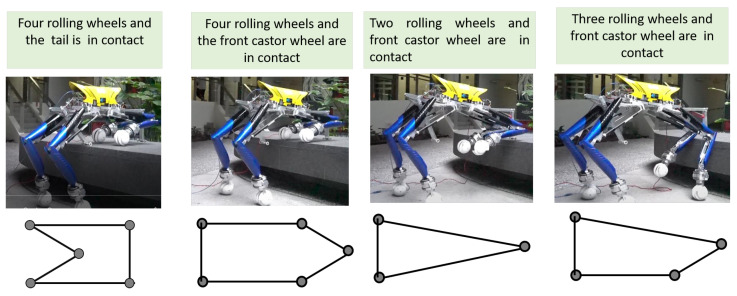
The support polygon for different gates of the platform during level shifting.

Due to a tiny perturbation or environmental disturbance, there is a high chance that the centre of gravity may fall outside the triangular area and cause instability to the platform. There is the need of a large support polygon to enhance the stability. Therefore, we incorporated a tail mechanism, two auxiliary wheels and an IMU based active correction to enhance the stability of the platform during level shifting. Figure 10 shows the different gait conditions for the Tarantula platform with locomotion wheels, auxiliary wheels, and a tail mechanism during level shifting.

**Figure 11:**
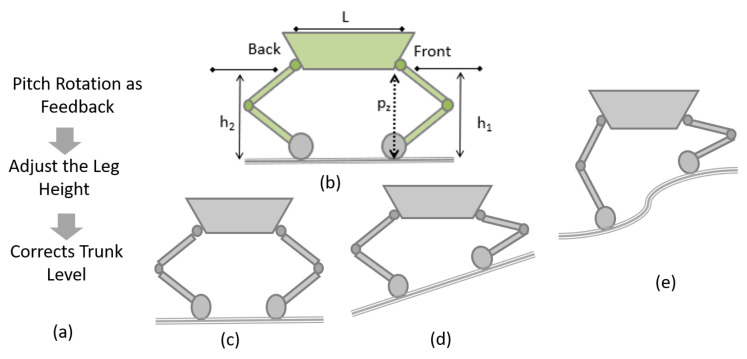
(**a**) Steps for trunk level correction. (**b**) Schematic of leg height. (**c**–**e**) Stable positions of the platform on different terrain conditions.

Pitch rotation of the platform leads to falls. To avoid platform falls, the pitch rotation of the platform is required to be controlled. While navigating, the platform must maintain a point of contact with the terrain and keep the trunk body horizontal as shown in Figure 11a. An IMU sensor is installed on the platform’s trunk, with the yaw axis along the robot’s height. The sensor helps to maintain the platform at a horizontal position at all times and keeps the centre of gravity at the same point.

On uneven terrain, inclined planes, or level shifting, the point of contact of each leg changes; hence, in order to maintain the posture, the height of each leg has to be adjusted. To balance the robot body (keeping the trunk at the horizontal position), the leg heights ($h_{1}$ and $h_{2}$) are to be adjusted. In addition, during level shifting, the objective is to keep the trunk in a horizontal position to avoid fall and keep the centre of mass always inside the projection point of contact on the horizontal plane as shown in Figure 11b–e.

**Figure 12:**
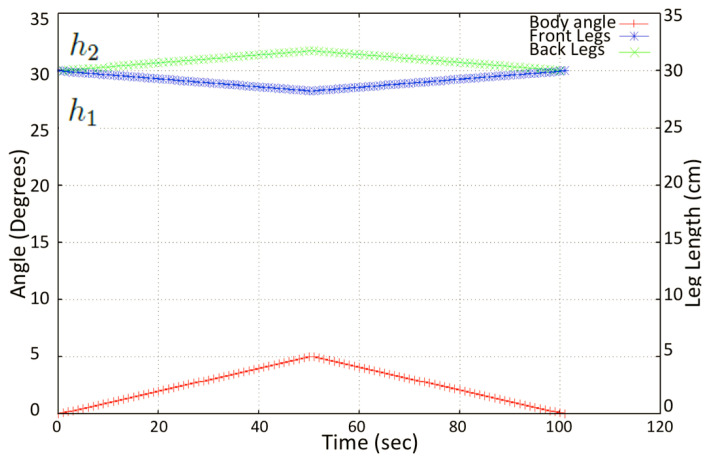
Variation of leg heights ($h_{1}$ and $h_{2}$ with respect to the trunk body (pitch angle) obtained upon simulation.

The height of each leg in terms of the abduction angle is simulated, and the variation of the leg heights are as given in Figure 12. During the transition, the levels in the drain, the IMU sensor, which is attached to the robot’s trunk, monitors the pitch angle, which is fed to a PID controller. The error signal from the controller is used to actuate the necessary actuators to maintain the stability of the robot by keeping the pitch angle in the range of −10 to +10 degrees.

## 5. Fuzzy Logic System for Wall Collision Avoidance

The objective of this subsystem is to maintain the robot in the middle of the drain to avoid possible collisions with the sidewalls during navigation. The robot needs to maintain its position in the middle with a small tolerance since the space availability of the drains is confined. The floor surface of drains is not smooth. Hence, there would be external disturbances for the robot, which causes unexpected lateral movements, if the robot was not controlled by perceiving the environment. As a solution to this problem, a fuzzy logic system is used for controlling the navigation of the robot.

Fuzzy logic is an intelligent method that can cope with a system with unknown dynamics or an exact process model [63,64]. It can model any complex behaviour that could be expressed linguistically by non-linear mapping between input spaces and output spaces [64,65]. Moreover, fuzzy logic systems can be considered as universal approximators [66]. In the case of Tarantula, the dynamic model changes with the reconfiguration of the robot. For example, the variation in the height of the robot changes the dynamic parameters of the robot. Thus, it would not be straightforward to have the exact dynamic of the robot in a situation.

In contrast, fuzzy logic systems have the ability to perform well without knowing the accurate underlying dynamics of a process or a system [67]. Furthermore, the sensory information of the robot about positioning is imprecise due to the rough surface characteristics of the walls of drains. Fuzzy logic can effectively manage its controlling actions even with imprecise sensory information [63,68]. In addition to that, fuzzy logic has been proven to be successful in coping with the navigation of robots in unknown environments [69,70,71]. Therefore, fuzzy logic is used to implement the subsystems for avoiding collisions with the sidewalls of drains during navigation.

**Figure 13:**
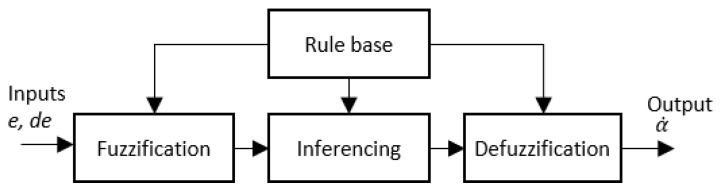
The architecture of the fuzzy logic system.

**Figure 14:**
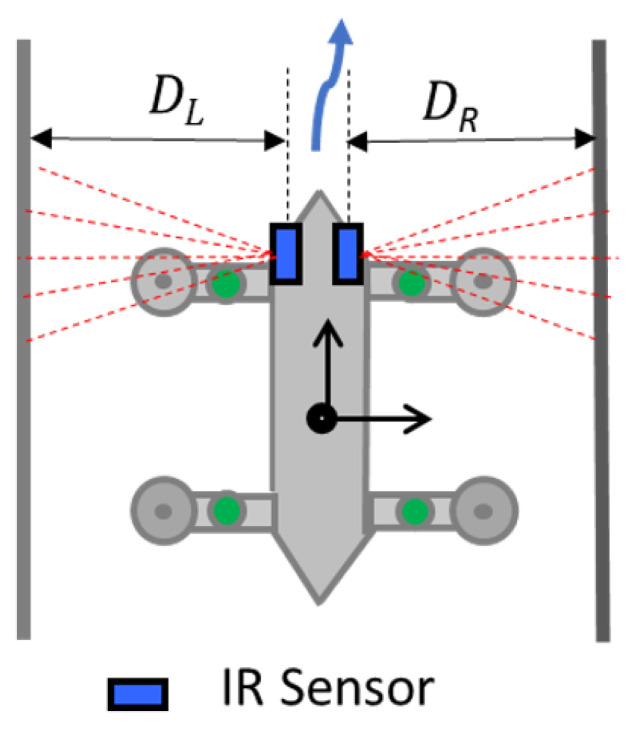
Perceiving $D_{L}$ and $D_{R}$.

The proposed fuzzy logic system is represented as a block diagram in Figure 13. The actual positioning of the robot is perceived as the distance from the robot to the left side wall and the right side wall ($D_{L}$ and $D_{R}$, respectively). These readings are taken from the distance sensors mounted on each side of the robot as shown in Figure 14. When the robot is in the middle of a drain, the distance to the sidewalls should be equal ($D_{L}=D_{R}$).

Thus, the error of positioning is estimated as $e=D_{L}-D_{R}$. The error of the position, *e* and the difference of the error, de (where de(t)=e(t)−e(t−1)) are taken as inputs to the fuzzy logic system to make the required control actions. These two inputs, *e* and de are measured in centimetres. The time step used for calculating de, (i.e., *t*) is 0.555 s. These two parameters reflect the present status and the trend, respectively. The present status and the trend of error are important for avoiding a possible collision with a side wall. These considerations are the reasons for selecting these two parameters as the inputs of the fuzzy logic system.

To avoid a possible collision, the robot’s action that should be controlled is the reference angular velocity of the robot, which eventually controls the angular velocities of the wheels. Thus, the output of the fuzzy logic system is the required reference angular velocity of the robot (i.e., $\dot{\alpha }$). The corresponding angular velocities of the virtual left and right wheels ($\omega_{L}$ and $\omega_{R}$) are determined with the aid of the kinematic model of the robot, and the wheels are controlled accordingly through their low-level controllers.

**Figure 15:**
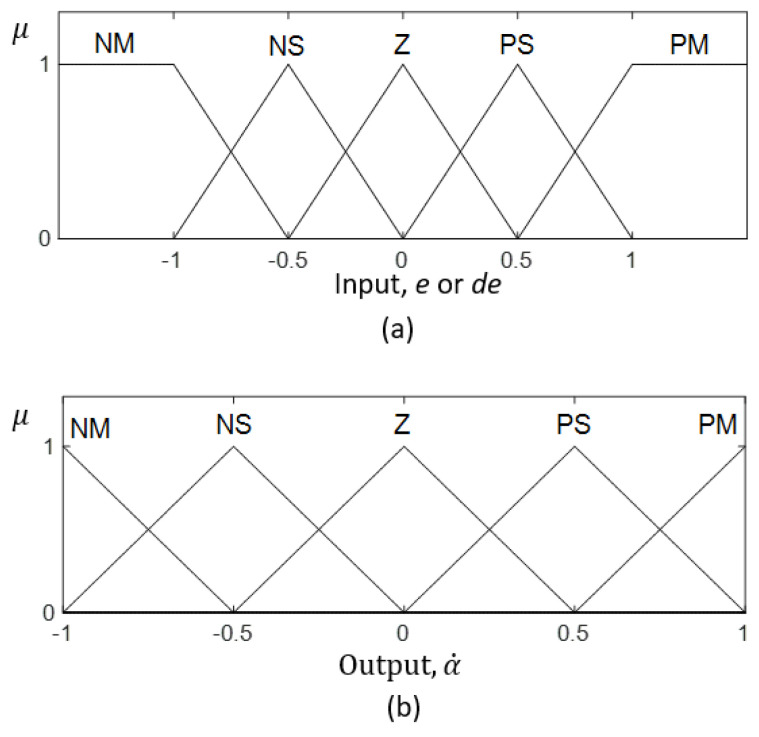
Membership functions of the fuzzy inference system. (**a**) The input membership functions. (**b**) The output membership function. The fuzzy labels are defined as NM: Negative Medium, NS: Negative Small, Z: Zero, PS: Positive Small, and PM: Positive Medium. The membership functions are given in normalized scales.

The two inputs, *e* and de, are fuzzified in the fuzzification layer. The fuzzification of the inputs is performed based on the corresponding input membership functions shown in Figure 15a. Each membership function consists of three triangular and two trapezoidal fuzzy sets. Triangular and trapezoidal membership functions require the least computational power compared to the other counterparts [72]. Thus, triangular and trapezoidal fuzzy sets were used for the sake of simplicity and efficiency of system implementation in a microcontroller.

The ranges of fuzzy sets were defined to uniformly cover the universe of the discourse of input and output spaces. The required number of fuzzy rules is increased with the number of fuzzy sets in input and output membership functions. The primary motivation of fuzzy logic is the use of human expert knowledge in modelling a controller through if–then linguistic rules. According to [73], the number of fuzzy sets in a membership function should be limited to seven for better representation of the linguistical meaning and efficiency.

Furthermore, the computational burden is also increased with the addition of rules and fuzzy sets. Based on these considerations, the number of fuzzy sets in a membership function was decided to be five, where the inputs and output space is covered with adequate sensitivity for this application. The corresponding fuzzified inputs can be considered as $\mu_{e}$ and $\mu_{de}$, respectively, for *e* and de.

A set of if–then rules that specify the necessary control actions of the robot is stored in the rule base. The fuzzy rule base of the proposed fuzzy logic system is given in Table 1. The rule base was heuristically defined based on expert knowledge. During the inferencing, the input fuzzy sets are mapped with the output fuzzy sets with the aid of this rule base. The output membership function is shown in Figure 15b. The fuzzy logic system considers the minimum and maximum as the fuzzy t-norm and t-conorm, respectively. Thus, the firing strength of the *i*th rule, $f_{i}$, can be expressed as in (4).

**Figure 16:**
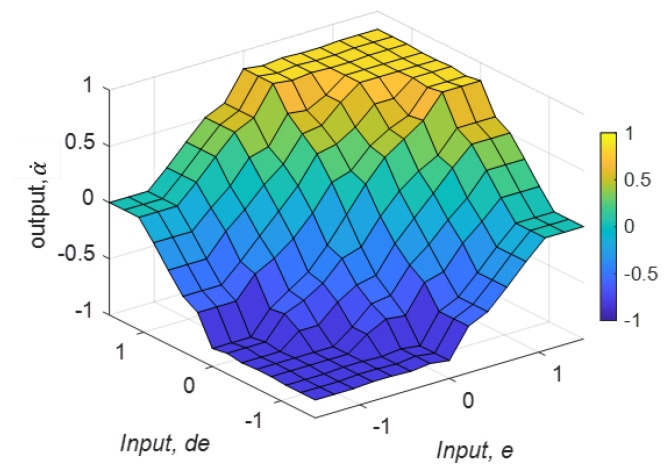
The decision surface of the fuzzy logic system.

Mamdani’s implication rule leads to the fuzzy consequent of the *i*th rule, $\mu_{\dot{\alpha }’}$ given in (5). The aggregated fuzzy consequents can be obtained from (6), where *n* is the number of rules. The aggregated set of output fuzzy consequents is defuzzified in the defuzzification layer. The defuzzified crisp output, ${\dot{\alpha }}_{0}$ can be obtained from (7). The respective velocities and the steering angles of the wheels are calculated in accordance with ${\dot{\alpha }}_{0}$ based on the kinematic model. The decision surface of this fuzzy logic system, where it reveals the variation of ϕ with respect to *e* and de, is presented in Figure 16.

(4) $$f_{i}=\mu_{e_{i}}\wedge \mu_{de_{i}}$$

(5) $$\mu_{{\dot{\alpha }}_{i}^{\prime }}=f_{i}\wedge \mu_{\phi_{i}}$$

(6) $$\mu_{{\dot{\alpha }}^{\prime }}=\mu_{{\dot{\alpha }}_{1}^{\prime }}\vee \mu_{{\dot{\alpha }}_{2}^{\prime }}\vee ...\mu_{{\dot{\alpha }}_{n}^{\prime }}$$

(7) $${\dot{\alpha }}_{O}=\frac{\int_{-\infty }^{\infty }\dot{\alpha }\mu_{{\dot{\alpha }}^{\prime }}d\dot{\alpha }}{\int_{-\infty }^{\infty }\mu_{{\dot{\alpha }}^{\prime }}d\dot{\alpha }}$$

## 6. Experimental Results and Discussion

### 6.1. Level Shifting Capability Using IMU

We tested the stability of the platform and the robustness of the adaptive control mechanism. As a test for the robot’s balance control algorithm with respect to the inclination of the terrain, the inclination was modified in a controlled way for four different cases: $0^{\circ }$ to $5^{\circ }$, $-5^{\circ }$ to $0^{\circ }$, $-10^{\circ }$ to $0^{\circ }$, and $0^{\circ }$ to $10^{\circ }$. Using the kinematics equations, the flexion-extension of the legs was controlled during height adjustment ($h_{1},h_{2}$), so that the body was always at $0^{\circ }$ of inclination even when the floor of the drain had variations in the inclination. We verified the trunk body balance using the IMU’s pitch angle for different tilt angles.

**Figure 17:**
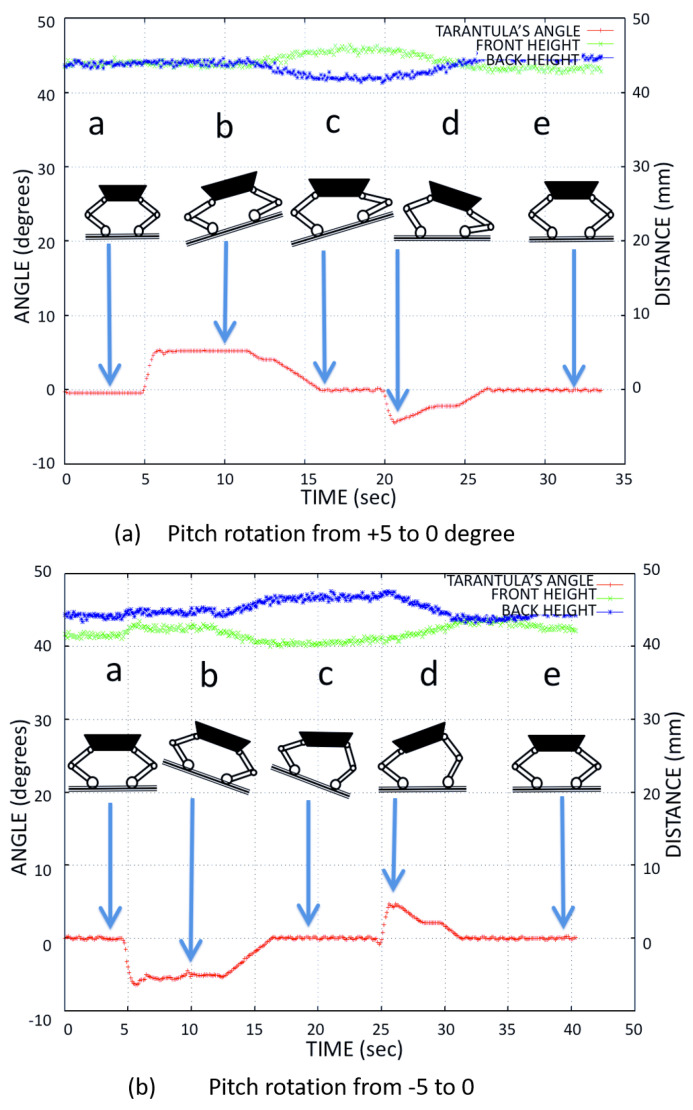
The Tarantula leg heights used for body balance during (**a**) $5^{\circ }$ and (**b**) $-5^{\circ }$ tilt angles of the trunk.

**Figure 18:**
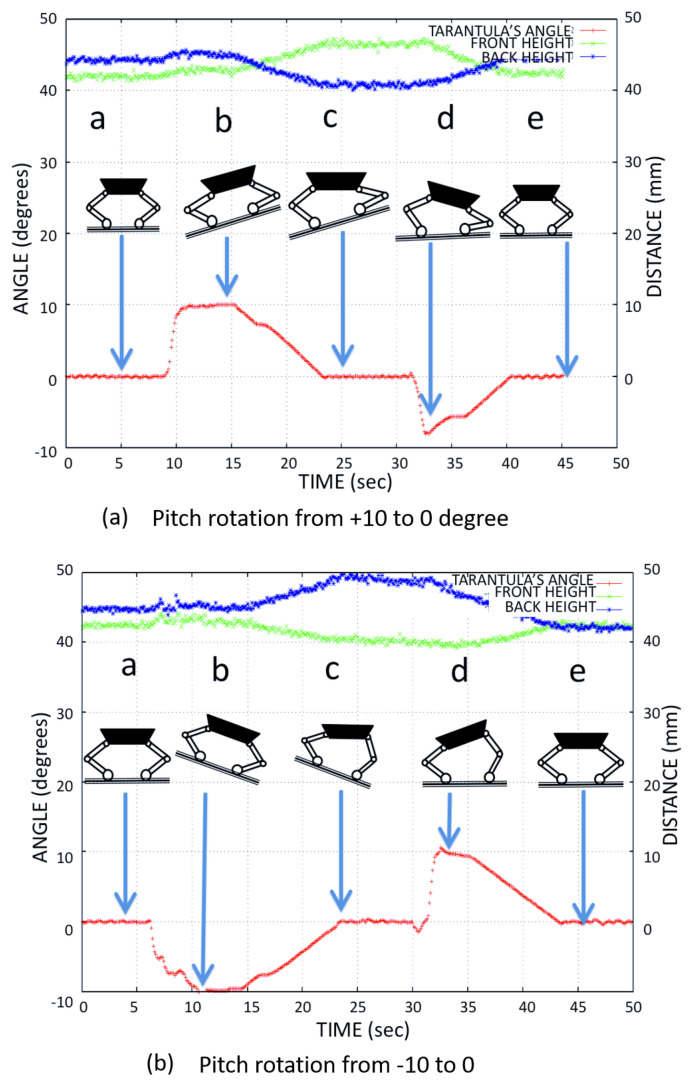
The Tarantula leg heights used for body balance during (**a**) $10^{\circ }$ and (**b**) $-10^{\circ }$ tilt angles of the trunk.

Figure 17 shows the adjustment of the front and back leg heights (the distance from the contact point to hip joint) when the platform balanced the body for the tilt angle $-5^{\circ }$ to $5^{\circ }$. Figure 18 shows the adjustment of the front and back leg height (distance from the contact point to the hip joint) when the platform balanced the body for the tilt angle $-10^{\circ }$ to $10^{\circ }$. The temporal variation of the pitch angle and leg height was close to the simulated result as shown in Figure 12.

**Figure 19:**
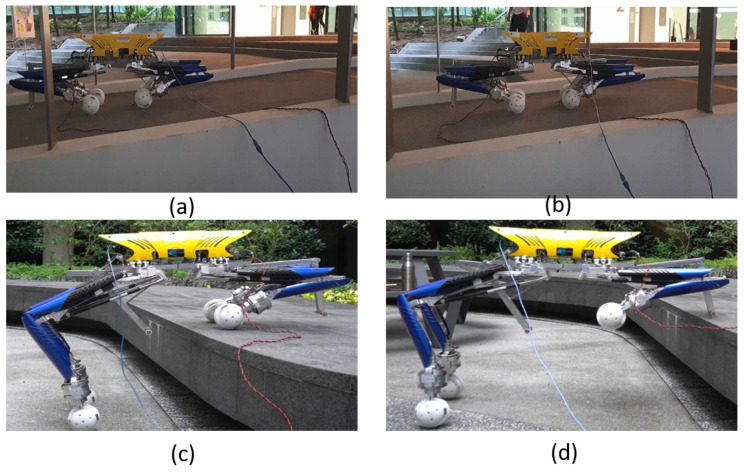
The platform’s pose with the trunk maintaining a horizontal position and leg height adjustment on (**a**,**b**) an inclined plane, (**c**,**d**) during a level shift.

We accomplished multiple trials for all the above tilt angles. For all these tilt angles, we accomplished multiple trials for each experimental test result. Figure 19 shows the platform’s locomotion on inclined terrain and the platform’s pose during level shifting with the trunk maintaining a horizontal position.

### 6.2. Wall Collision Avoidance Based Navigation

The Tarantula was tested in different circumstances in both laboratory settings and real drain environments to verify the performance of the fuzzy logic system implemented. First, a virtual drain environment of 4 m in length was created by setting up walls at a distance of 80 cm. The Tarantula was placed in between the walls and made to move autonomously over the length of the virtual drain. The Tarantula was successful at moving over the length of the drain without hitting the walls.

**Figure 20:**
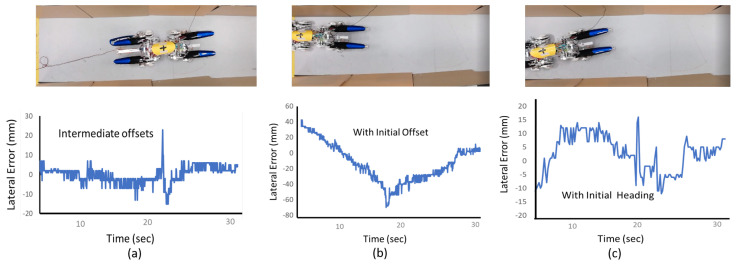
Wall following experiments for an (**a**) intermediate offset, (**b**) initial offset, and (**c**) initial heading error of the wall.

This was tested in various cases, including the Tarantula starting with an initial heading offset, a lateral offset during the initial setting (lateral displacement from the centre), and with an intermediate offset of one of the walls (to simulate the uneven nature of the wall surfaces of drains), as shown in Figure 20. In each case, the robot managed to move back to centre of the virtual drain without hitting the walls with fuzzy logic control. Thus, these experimental results validate that the fuzzy logic system can correct the lateral positioning of the robot.

The response of the controller appeared slow in the case shown in Figure 20b. This slowness could have been avoided by configuring the system to have a more aggressive response in changing the robot’s angular velocity. On the other hand, the robot’s aggressive turning would increase the collision of the robot’s back to the sidewall (opposite to the turning direction) since the robot has a considerably high length. The primary motivation of the proposed controller is to avoid possible collisions and improve the robot’s safety.

Thus, the controller performance, such as the response speed, must be balanced without compromising safety. Furthermore, this case represents what is likely a maximum error scenario since the robot was placed close to the sidewall. The results of this test case indicated the controller’s ability to correct an initial lateral error without compromising the safety of possible collisions.

**Figure 21:**
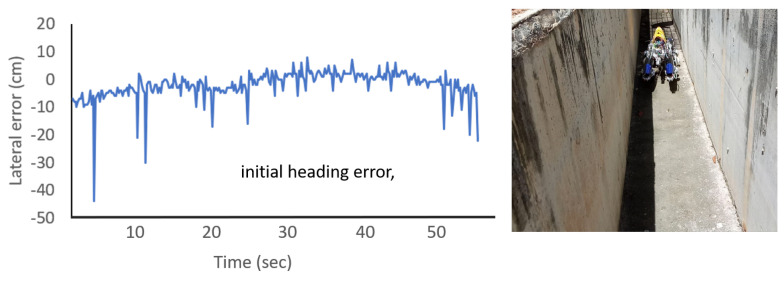
The collision avoidance results in a real drain.

Similarly the collision avoidance ability of the robot was tested in a real drain environment. The robot managed to move across the length of the drain without hitting walls. The variation of the lateral positioning error perceived by the robot through the sensors is shown in Figure 21. The proposed fuzzy logic system was able to rectify the positioning errors caused due to external disturbances, such as rough surfaces. The locomotion of Tarantula in the drain was not as smooth as in the virtual drain because of the very rough surface in the real drain. Nevertheless, the robot was able to navigate without colliding with the walls.

Moreover, the controlling action of the robot was sufficient for avoiding collisions. Sudden spikes in error can be observed in the plot. These error spikes are due to the noise of the sensors caused due to the rough characteristics of the wall surface. The use of an additional filtering technique could overcome these sensor noises. On the other hand, fuzzy logic itself has immunity to sensor noises and has the ability to cope well with them. Therefore, an explicit filtering method was not used for the distance measurements. We hope to examine the possible use of filtering methods for improving the controller performance in the future.

## 7. Conclusions

In addressing the challenge of inspecting drains that have multiple level shifts and varying heights, we designed and deployed a self-reconfigurable robot in an actual drain environment. In this paper, the following objectives of the developed drain mapping robot were accomplished: (1) The variable height of the robot was achieved using rolling wheels and linear actuator-based legs. (2) An adaptive algorithm controller with IMU-based feedback was developed to maintain the trunk body horizontally irrespective of the drain height and terrain conditions. (3) A fuzzy logic system that analysed the readings of IR sensors was developed to help the platform to avoid collisions with the side walls of the drain. The proposed level shifting and wall collision avoidance system design were implemented into the real system, and the experimental results demonstrated the effectiveness, level shifting capability, and auto correction to maintain a safe distance from the wall of the platform.

Typically, a drain surface is cemented and, thus, a rugged terrain, which makes it difficult for a robot to move smoothly. These difficult conditions are further escalated due to debris, such as leaves, gravel, and water. Thus, the provided results represent the behaviour of the robot on a portion of the targeted conditions. However, experiments were not conducted on test environments with a flow of water, since the robot has not yet been developed considering waterproofing. For future work, we expect to conduct experiments in situations where there are water flows.

## Figures and Tables

**Table 1:** Rule base of the fuzzy logic system.

de\*e*	NM	NS	Z	PS	PM
NM	NM	NM	NM	NS	Z
NS	NM	NM	NS	Z	PS
Z	NM	NS	Z	PS	PM
PS	NS	Z	PS	PM	PM
PM	Z	PS	PM	PM	PM

## Data Availability

No new data were created or analyzed in this study. Data sharing is not applicable to this article.
